# Introducing a Computerized Figural Memory Test Based on Automatic Item Generation: An Analysis With the Rasch Poisson Counts Model

**DOI:** 10.3389/fpsyg.2020.00945

**Published:** 2020-06-10

**Authors:** David Jendryczko, Laura Berkemeyer, Heinz Holling

**Affiliations:** ^1^Institute for Psychology, Universität Konstanz, Konstanz, Germany; ^2^Institute for Psychology, Westfälische Wilhelms-Universität Münster, Münster, Germany

**Keywords:** figural memory, short-term memory, visual information load, parallel test forms, automatic item generation, item response theory, Rasch Poisson counts model

## Abstract

An automatic item generator for figural memory test items called *figumem* was developed. It is available in *R*. A cognitive model allowed the generation of hypothetically parallel items within three difficulty levels determined by visual information load. In a pilot study, participants solved three items for each level of visual load. Within an item response theory approach, the Rasch Poisson counts model and modifications of it were fitted to the data. Results showed overall satisfying fit. Visual information load explained most of the variance in item difficulty. Differences in difficulty between items of the same family were comparatively low, displaying the utility of the item generator for the creation of parallel test forms. Implications, limitations, and suggestions for the use and extensions of figumem are discussed.

## Introduction

Automatic item generation (AIG) is a modern approach for developing items and tasks, especially for tests measuring cognitive abilities. It is usually based on theoretically and empirically validated quality control mechanisms. Thus, it avoids misinterpretations in item writing by human item writers and facilitates an enhanced interpretation of test scores (Lai et al., 2009). If the generating rules are known, AIG can be used to automatically produce large numbers of high-quality items. AIG is the computer algorithm–controlled creation of tasks/items under a predefined item prototype called *item model* (Gierl and Lai, 2012). This means that computer algorithms are used instead of conscious decisions to generate families of items from a smaller set of *parent items* (Glas et al., 2010).

The construction of an item model can be enriched if it is based on a theory that predetermines the level of item difficulty, among other measurement properties (Irvine, 2002). *Radicals* (Irvine, 2002) are structural elements that significantly affect item parameters (such as item difficulty) and provide the item with certain cognitive requirements. One or more radicals of the item model can be manipulated in order to produce parent items with different difficulty levels. Each parent can then grow its own *item family* by manipulating other elements that Irvine called *incidentals*. Incidentals are surface features that vary randomly from item to item within the same family. Items that have the same structure of radicals and only differ in incidentals are usually labeled *isomorphs* (Bejar, 2002) or *clones* (Arendasy and Sommer, 2012). The variation of items’ surface characteristics should not significantly influence the testee’s responses, which is the reason why it is believed that incidentals produce only slight differences among the item parameters of the isomorphs.

Automatic item generation so far has mostly been used for the construction of reasoning tests with figural (e.g., Bejar, 1990; Embretson, 2002; Arendasy and Sommer, 2005, 2010; Gierl et al., 2008; Zeuch et al., 2011; Bertling, 2012; Blum et al., 2016; Loe, 2019), numerical (e.g., Arendasy et al., 2006; Gierl et al., 2008; Holling et al., 2009; Arendasy and Sommer, 2012; Bertling, 2012; Loe et al., 2018; Loe, 2019) and verbal (e.g., Gierl et al., 2008; Loe, 2019) material but was also employed for the measurement of other cognitive abilities, comprehensively described in the Berlin intelligence structure model (BIS; Jäger et al., 2006). In this model, the four cognitive traits of reasoning, mental speed, creativity, and memory are conceptualized to operate with figural, numerical, and verbal contents for a measurement of general intelligence (or psychometric g). Doebler and Holling (2016) presented test generators for mental speed with all of these contents of stimulus material. Generators for creativity and memory have received less attention. Regarding the former, the measurement of creativity *per se* is problematic, as it is difficult to clearly define the cognitive operations at hand when new ideas are produced. Moreover, the response format is usually open, and the determination of test scores requires trained raters (see Forthmann et al., 2019c). Regarding the latter, the lack of AIG-based item generators for short-term and long-term memory items presents a void in the scientific literature that demands to be filled.

Automatic item generation has a number of benefits, especially compared to traditional test creation (see Bertling, 2012). If the item model and its cognitive basis are properly articulated and the stimulus material carefully selected, then items are created quickly, efficiently, and with comparatively low cost. A test of the item model can simultaneously be considered a quality control for the item generator and a check for construct validity of the proposed cognitive ability. One major drawback of fixed sets of traditionally created items is that they cannot be used once they have been exposed to the public. People then have the opportunity to learn the correct solutions prior to the assessment instead of actually engaging in the test tasks during the test session. This problem is obviously magnified in an increasingly interconnected world. One example for this from the memory domain is given with the Rey–Osterrieth complex figure test (Rey, 1941). The figure has been depicted in various textbooks (e.g., Banich and Compton, 2018, p. 210; Kolb and Whishaw, 2015, p. 419; Schellig et al., 2009, p. 527) and can be looked up with an online search engine.

One particularly important advantage of AIG is that if an item model for the creation of test items can be properly defined, a multitude of parallel items, which differ in their presentational features but are identical (or at least very similar) in their psychometrical properties (such as difficulty), can be created. Parallel test versions are often required in longitudinal studies and diagnostic assessments to separate actual temporal improvements of the cognitive ability from mere retest effects emerging from repeated exposure to the same test items (see Reeve and Lam, 2005; Jendryczko et al., 2019). Alternative versions for short-term memory (STM) items are essential, as repeatedly presenting the same items to testees will confound their STM performance with effects of long-term memory retrieval. This is especially relevant for various clinical populations, as an ongoing decay in memory functions due to a disease or disorder must be validly measured and documented. The same applies to improvements of memory functions due to an intervention. In this regard, a multitude of automatically generated items can also effectively be used for training purposes. Examples from clinical neuropsychology, where some or all of these benefits apply, are given with syndromes, disorders, or diseases in which learning of new information is impaired, such as Korsakoff syndrome (Lloyd et al., 2019), Alzheimer’s disease (Liang et al., 2016), Huntington disease (Clemensson et al., 2017), Parkinson’s disease (Rolinski et al., 2015), anterograde amnesia (Stöllberger et al., 2019), and post-traumatic amnesia (De Simoni et al., 2016).

Many of the commonly used STM tests have few or no parallel test forms. For example, the internationally most often-used memory test battery (Thöne-Otto, 2009), the Wechsler Memory Scale—Revised (WMS-R; Bornstein and Chelune, 1988), currently holds no alternative test version. Similarly, commonly applied visual STM tests as the Benton-test (Benton et al., 1996), the Rey–Osterrieth complex figure test (Rey, 1941), the non-verbaler Lerntest (Eng.: non-verbal learning test; NVLT; Sturm and Willmes, 1999), the recognition memory test (Warrington, 1984), and the doors test (Baddeley et al., 1994) either have no parallel versions at all, or their true psychometrical equivalence is questionable, as respective studies are missing (Thöne-Otto and Markowitsch, 2004; Schellig et al., 2009; Thöne-Otto, 2009). This might not be surprising, given that alternatives to complex, figural materials are more difficult to create and implement digitally compared to alternative words or numbers. Two positive examples for visual memory tests delivering alternative test forms are the Lern- und Gedaechtnistest-3 (Eng.: learning and memory test-3; LGT-3; Bäumler, 1974), which has a total of three equivalent test versions, and the Visueller und Varbaler Merkfaehigkeitstest (Eng.: visual and verbal memorizing ability test; VVM; Schellig and Schächtele, 2001), that offers four parallel forms. For an overview of commonly applied memory testing procedures, see Schellig et al. (2009).

### Research Purpose

The purpose of the current work is to introduce “figumem,” a new computerized test for figural memory based on AIG that is readily available to researchers and practitioners in the free software “R” (R Core Team, 2018). The test itself and a user’s manual are available as online Supplementary Material to this article.

We focused on figural STM (although the test may be useful for the assessment of long-term memory as well, as explained in the section “Discussion”), because the lack of parallel visual memory tests is particularly prominent and because the use of figural test material is less dependent on the testee’s language skills and mathematical knowledge. We display a theoretical item model based on the *visual information load* phenomenon (Alvarez and Cavanagh, 2004) to introduce figural memory tests into the AIG approach. We report the results of a study in which the item model was probed in the frameworks of item response theory. In that context, we explain the utility of the Rasch Poisson counts model (RPCM; Rasch, 1960/1980) for a statistical representation of our item model.

The rest of the paper is structured as follows: We introduce the item-generation model specified for figumem. We then proceed to illustrate the RPCM. Finally, we present an empirical study that incorporates the RPCM to examine the statistical properties of the item generator.

### A Cognitive Model for the Automatic Generation of Figural Short-Term Memory Items

The distinction between a visual and an auditory STM remains a largely undisputed concept across various memory models. The Baddeley model of working memory (WM; Baddeley and Hitch, 1974; Baddeley, 1978, 1992) was among the first models to present a clearly articulated relation of these sensory modalities to memory functions. While the details of the underlying processes are still subject to current research, the model still serves as a general framework (see, for example, Kosslyn, 2005; Baddeley et al., 2011; Logie, 2011; Baddeley, 2012; Slana Ozimič and Repovš, 2020). In order to avoid confusion, it should be stressed that “the memory system or systems responsible for STM form part of the *working memory* system” (Baddeley et al., 2015, p. 41). This means that the assessment of STM differs from that of WM only in the type of the presented task. Whereas the former merely requires learning of stimulus material for immediate or only slightly delayed recall, the latter additionally requires manipulation of the learned material. Consequences of the type of stimulus material and the modality it is processed with apply to both memory systems.

The model contrasts a visuospatial sketchpad of memory responsible for the maintenance and manipulation of visual information from the phonological loop that coordinates processing of audible information. Research showed that, generally, human beings are able to phonologically hold numbers and verbal material in their STM without affecting the processing of visual material and vice versa (e.g., Sanders and Schroots, 1969; Logie et al., 1990; Duncan et al., 1997; Cocchini et al., 2002). Figural memory tests are used to assess the visuospatial sketchpad, specifically.

Fundamental research on the functionality of STM has focused on memory capacity (e.g., Luck and Vogel, 1997; Vogel et al., 2001; Wheeler and Treisman, 2002; Alvarez and Cavanagh, 2004; Allen et al., 2006). In other words, researchers investigated the number of units of information human beings can hold in their STM. It became clear that the term “units of information” is difficult to unambiguously define in general and for visual material in particular. Alvarez and Cavanagh (2004) found that the capacity of visual STM is determined by not only the number of visual objects but also the visual information load (or just “visual load”) each individual object holds. In their conception, visual information load is given by the amount of visual detail in a stimulus and is operationalized with a visual search task. The more the objects load visually, the longer it takes to identify these objects among other similar objects, and the less objects can be stored in visual STM. Importantly, Ueno et al. (2010) and Ueno et al. (2011) later demonstrated that visual information load of an object can at least partially be determined by bindings of stimulus features (e.g., shape and colors), as long as this increases the similarity of the objects (see also Jiang et al., 2009). To give an example from Alvarez and Cavanagh’s experiment: A square with a certain color in an array of differently colored squares of the same size can be found relatively quickly because these stimuli are all distinctively defined by the single feature of color. In contrast, finding a three-dimensional cube with a certain orientation and a certain pattern of shadings along its visible surfaces among several differently oriented and shaded cubes takes comparatively longer. These stimuli contain more visual detail and less salient differences, as orientation and shading must be considered simultaneously for detection in the search task. The authors measured the search rate for different kinds of visual stimuli such as these and investigated the relationship between the average search rate and the average visual STM capacity for the respective objects. They found that STM capacity decreases for visually more loaded objects. Visual information load (operationalized as visual search rate) explained 99.2% of the variance in visual STM capacity.

This finding represents the base for the radical of our implemented item model. Its translation into the item format is explained in the next two sections.

### Item Format of Choice

Generally, tests that measure visual STM can be distinguished into tests that require the memorization of the spatial layout of a scene, such as a marked way through a maze (e.g., Schellig and Schächtele, 2001), and tests that require the memorization of specific figures or the association between specific figures (e.g., Bäumler, 1974). We refer to the former as spatial memory tests and to the latter as figural memory tests. Whereas both item formats require visual STM performance, it is debatable whether or not both formats actually measure the same trait. Findings from neuropsychological research converge into the separation of visual memory into a spatial and an object domain (Funahashi et al., 2004; Todd and Marois, 2004, 2005; Logie and Van der Meulen, 2008; see Wager and Smith, 2003, for a meta-analysis). We decided to design our item generator based on figural memory tests that incorporate figural associations. In such tests, testees have to memorize which figure from a pool A was presented alongside which figure from a pool B. We made this decision for various reasons. The most obvious one is that our cognitive framework specifically stresses the importance of visual load of figures instead of spatial layouts. A transfer to spatial layouts is not straightforward. Moreover, attempts at AIG of spatial memory items have already been made (Hornke, 2002; Arendasy et al., 2008), rendering the demand for an automatically item-generating figural memory test more contemporary. Finally, tests that require the memorization of figural associations offer a certain flexibility regarding the answer format. The tester can demand a reproduction (that is, a drawing) of a figure that was associated with a different figure during the memorization phase (open response format). This is, however, more difficult to implement into online surveys and hence unpractical if one aims at an efficient and fast recruitment of study participants. Alternatively, researchers can use a closed recall format in which the testee has to choose the correct figure from a selection of figures. This is convenient for online testing procedures but increases the probability of guessing the correct answers. Figumem was constructed with the latter format in mind, but alternatives corresponding to a free answer format are already implemented and elaborated on in the section “Discussion” and in the test manual.

**FIGURE 1:**
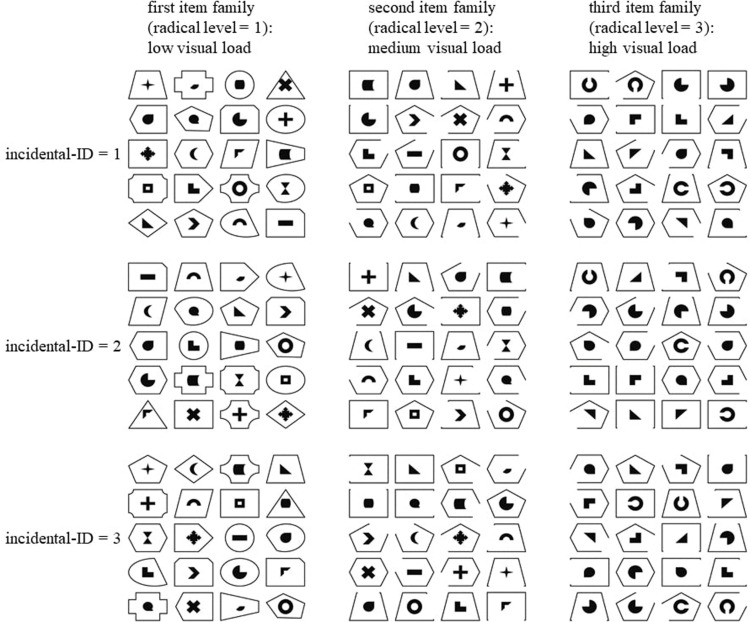
The nine figumem items that were incorporated into the current study. The Supplementary Material to this article contains a script for the replication of these items with figumem. The radical of visual load determines item difficulty (increasing item difficulty with increasing magnitude of visual load). For each condition of visual load, three items were produced. They are differentiated by different (randomly occurring) assignments of emblems to frames and by presentation order of the emblem–frame units along the 5 × 4 matrix. These different realizations on the incidentals are encapsulated by one index variable (incidental-ID). Understanding this figure as a 3 × 3 table of figumem items, item difficulty is expected to vary within the rows and to be constant within the columns.

The following concrete format for figumem items was determined: In every item, 20 emblems are presented to the testee in a 5 × 4 matrix. Each emblem is surrounded by a frame (see Figure 1 for examples). The testee has 1 min to learn which frame surrounds which emblem. After the time elapses, the emblems are presented again in a new screen in a 5 × 4 matrix but in a different order. Under each emblem, four frames are presented, of which one resembles the frame the emblem was surrounded by in the previous screen. The other three are distractors. The testee’s task is it to mark the correct frame for each emblem. The score for this task is given by the sum of correctly marked frames and can range between 0 and 20.

### Designing the Item Generator

We created 20 original emblems and 20 original frames as PNG data files. Additional stimulus material was produced by modifying some of the emblems and some of the frames. A radical with three different levels and incidentals were determined that govern the rules for combining the various stimuli for the creation of three different figumem parent items and their respective families. Figure 1 presents three example figumem items for every radical level (i.e., for every item family). This figure can ease the understanding of the following explanations.

The radical levels correspond to three different levels of visual load. Visual load itself increases with increasing similarity between the visual stimuli within an item by adding additional visual features. This in turn implies an increase in item difficulty. For items with low visual load, all 20 original emblems and all 20 original frames are used. They are all distinctively defined by the feature of figural shape. Hence, visual load is comparatively low, as only the feature of shape needs to be remembered to correctly replicate the associations between emblems and frames (examples in the first column of Figure 1).

For items with medium visual load, the use of the 20 original emblems is maintained. However, only four of the original frames are still present in items from this family: the rectangle, the trapeze, the pentagon, and the hexagon. For each of these, four additional variants were created by erasing one line of the respective frame, which results in 20 different frames in total again. The frames of items from this item family have more similarities to one another, as some frames have the same shape and can only be differentiated from each other by also considering the feature of the missing line (or completeness of lines). This increases visual load (examples in the second column of Figure 1).

For items with high visual load, the frames from the second item family are maintained. Only four of the original emblems are still used: the right triangle, the black circle with the missing quadrant in the top right, the black circle with the prong in the top right, and the “L”-shape. For each of these, three additional variants were created by rotating the emblem 90°, 180°, and 270°. This results in 16 emblems. Four additional emblems were created by taking the black ring from the original 20 emblems and editing out a piece at the top, at the right, at the bottom, and at the left. In items from this family, the increased similarities between the frames from the second item family are still present, but similarities between the emblems are additionally increased. To differentiate the emblems from each other, one has to simultaneously consider the features of shape and direction. This increases visual load even further compared to the first two item families (examples in the third column of Figure 1).

Three item presentation features were considered incidentals that can occur randomly: (1) the assignment of an emblem to a frame; (2) the order of the 20 emblem–frame units along the cells of the 5 × 4 matrix; and (3) the three frames that are used as distractors for each emblem in the closed answer format (not depicted in Figure 1). An important constraint was made so that each frame appeared equally often as a distractor in the closed answer format, since otherwise, test-specific strategies (exclusion of specific distractors) could determine the outcome of an item (see, for example, Millman et al., 1965; Tarrant et al., 2009).

Note that between the items within each row of Figure 1, the families and thus the radical levels, the employed stimulus material, and the hypothesized item difficulties differ. In contrast, within each column of Figure 1, all three items consist of the same stimulus material because they stem from the same item family. Only the incidentals of concrete emblem–frame units and presentation order are different^1^. The nine items from Figure 1 represent those that were incorporated into the current study. The Supplementary Material to this article contains a script for the replication of these items with figumem. Each item is uniquely identified by its radical level (or family; 1, low visual load; 2, medium visual load; 3, high visual load) and its level on a categorical index variable (incidental-ID) that encapsulates the item’s random realizations on the incidentals. The actual production of the visual material within figumem is accomplished by reading in the PNG data files containing the emblems and frames and then editing them with the “magick” software package (Ooms, 2018) in R.

In a nutshell, the difficulty of an item is determined by the radical of visual load. At the easiest difficulty level, all frames are identified by their shape, and the same applies to the emblems (low visual load). At an intermediate difficulty level, a different subset of frames is used, and these frames are only identifiable by simultaneously considering their shape and the completeness of lines or position of a missing line (medium visual load). At the highest difficulty level, a different subset of emblems is additionally used. These emblems are only identifiable by simultaneously considering their shape and direction (high visual load). For each item family, the particular emblem–frame combinations and their presentation order are considered incidentals. A constraint is made so that every frame appears equally often as a distractor in the closed answer format.

At this point, the item difficulty dependence on the radical and the statistical equivalence of isomorphs were only given on the hypothetical level. For an actual test of these hypotheses, the item model needed to be translated into a statistical model.

### A Statistical Representation of the Item Model

Based on the Poisson distribution, Rasch (1960/1980) proposed a model that predicts an item score (the sum of correctly remembered associations for an item in this case) with item and person parameters. This model is known as the Rasch Poisson counts model (RPCM). In this model, it is assumed that the probability for a score *Y*_**ν* i*_ of person ν on item *i* follows a Poisson distribution:

(1) $$P\left(Y_{\nu \,i}=y_{\nu \,i}\right)=\frac{e^{-\mu_{\nu \,i}}\,\mu_{\nu \,i}^{y_{\nu \,i}}}{y_{\nu \,i}!}$$

Here μ_**ν* i*_ reflects the expected score for person ν on item *i*. The crucial assumption within the RPCM is that this expected score is given with the product of a person’s ability parameter θ_**ν**_ and an item’s easiness parameter σ_*i*_:

(2) $$\mu_{\nu \,i}=\theta_{\nu }\,\sigma_{i}$$

The RPCM can be understood as a generalized linear model (GLM) with a log-link that contains persons and items as predictors:

(3) $$\log\left(\mu_{\nu \,i}\right)={\tilde{\theta }}_{\nu }+{\tilde{\sigma }}_{i}$$

with ${\tilde{\theta }}_{\nu }$ = log (θ_*ν*_) and ${\tilde{\sigma }}_{i}$ = log (σ_*i*_).

Because of the person parameters, the model holds the property of an increasing amount of parameter estimations with increasing sample size. In most cases (such as the current study) the item parameters are of primary interest, as the model is usually used to investigate the psychometric characteristics of a test. This property is, thus, disadvantageous because it also implies enlarged standard errors for item parameters when the joint maximum likelihood estimation method is used. For this reason, the model is usually estimated as a generalized linear mixed model (GLMM) using marginal maximum likelihood. Within this approach, person parameters (on the log-scale) can be conceptualized as realizations of a random intercept variable following a normal distribution (Doebler and Holling, 2016):

$${\tilde{\theta }}_{\nu }∼N\,\left(0,\text{ζ}\right).$$

The mean of this distribution can be fixed to zero for model identification, and only the variance (ζ) of the distribution needs to be estimated. Within this approach, σ_*i*_holds the convenient interpretation of the expected score on item *i* given average (cognitive) ability.

Item models in the AIG framework assert that the radicals determine item difficulty. In order to verify this claim, the item parameters themselves can be explained with a combination of radicals (Fischer, 1973; Zeuch, 2010). In figumem, only one categorical radical (visual load) with three possible outcomes is given that can be cell-mean coded for convenient parameter interpretations. Let *x*_*il*_ represent the condition *l* (with *l* = 1, 2, 3) of visual load (1, low; 2, medium; 3, high) that is active (*x*_*il*_ = 1) or inactive (*x*_*il*_ = 0) in item *i*. It follows that

(4) $${\tilde{\sigma }}_{i}=\eta_{1}\,x_{i\,1}+\eta_{2}\,x_{i\,2}+\eta_{3}\,x_{i\,3}$$

and

(5) $$\log\left(\mu_{\nu \,i}\right)={\tilde{\theta }}_{\nu }+\eta_{1}\,x_{i\,1}+\eta_{2}\,x_{i\,2}+\eta_{3}\,x_{i\,3}.$$

Here *e*^η*l*^ represents the expected item score on items with radical level *l* given average ability.

If the RPCM (regardless whether the items themselves or the radicals are used as predictors) fits, it should be used because it holds some of the same statistical properties as the widely recognized Rasch 1PL model for dichotomous data (see De Ayala, 2009; Eid and Schmidt, 2014). The sum of the item raw scores serves as a sufficient test statistic, and specific objective comparisons between persons and between items can be made. In the following, we describe an empirical study in which we investigate the fit of the RPCM for tests created with figumem. We determine whether the radical sufficiently explains item difficulty and evaluate the variability of item difficulty within item families. In the section “Analytic Strategy,” we describe how we make use of the models listed here for these purposes.

## Materials and Methods

### Sample

Participants were recruited via social network services that reach out to university students with scholarships and members of the academic club CdE e.V. and via flyers at the university of Münster and at the university of Konstanz. A total of 234 examinees completed the online survey. 26 participants had to be excluded because they reported that their screen did not always display the complete item. The final sample consisted of *N* = 208 participants (146 reported to be female, 61 reported to be male, 1 person did not state his or her gender). The mean age was 22.51 years (SD = 6.18). One hundred eighty-one examinees were university students, among which 123 studied psychology. 11 participants were secondary school students, and 16 participants already worked in their professions. The psychology students had the opportunity to gain course credit for their participation. The remaining examinees participated as a favor to the second author.

### Material

In order to evaluate an automatic item generator, as many items as possible from each item family should be presented to participants. This number will, however, always be constrained by reasonability considerations. Figural memory items exhaust concentration and cognitive resources rather rapidly. During the pre-study testing phase, we came to the conclusion that participants should not be required to work on more than nine figumem items in one session.

**FIGURE 2:**
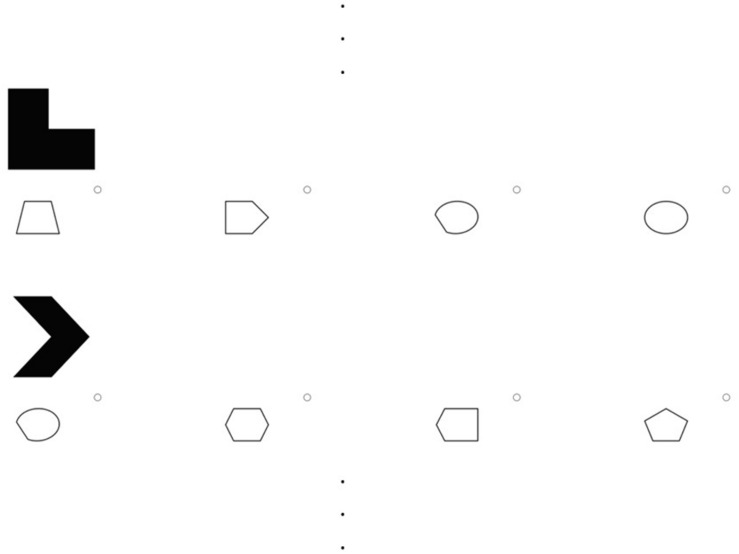
Example of the answering format for figumem items implemented in the current study. In contrast to the originally conceptualized format, the emblems and their respective option pools for frames are not presented in a 5 × 4 matrix but one below the other in a random order. The three dots at the top and at the bottom indicate the rest of the 20 emblems and their pools of frames of a figumem item that are not depicted. The item generator can produce both this and the original format (see the manual from the Supplementary Material).

A total of 12 figumem items (4 for every difficulty level) were generated. These items were implemented into the online-survey software *Questback*. One item of each difficulty level served as a mere example item for illustration purposes; the other nine (see Figure 1) were used for data collection. It was not possible to implement our original answering format for the retrieval phase into Questback. Instead of being displayed in a 5 × 4 matrix, emblems were presented one below the other with four options for frames arranged underneath each of them (see Figure 2). The order of emblems was random. Testees had to click on the frame of choice for one emblem and scroll down to make the decision for the next emblem. Both the original answering format and this format are available in figumem (see the manual from the Supplementary Material).

The link to the study was distributed to the participants. It was ensured that the survey runs smoothly in every mainstream internet browser.

### Procedure

Upon clicking on the study link, participants were directed to the first site of the survey. Here they were greeted and informed about the length of the test and the opportunity for psychology students to gain course credit. Furthermore, participants were informed about anonymity, voluntariness of participation, and their right to cancel participation at any time. Informed consent was asked for. Next, participants were requested to concentrate during the test and to abstain from the usage of supporting devices like paper and pencil. The survey went on to present participants a black rectangle in the size of a figumem item. Participants were asked only to proceed with their monitor fully displaying this rectangle. They were given the chance to switch to a different device if this condition was not fulfilled.

Afterward, the procedure of the figural memory test was explained, and one example item for every difficulty level was shown. Practice material with only two emblems and two frames was presented. On the next page, the two emblems with a selection of four frames each were given for familiarization with the answering format. Participants had to click on the correct frames to select them as their answers.

**FIGURE 3:**
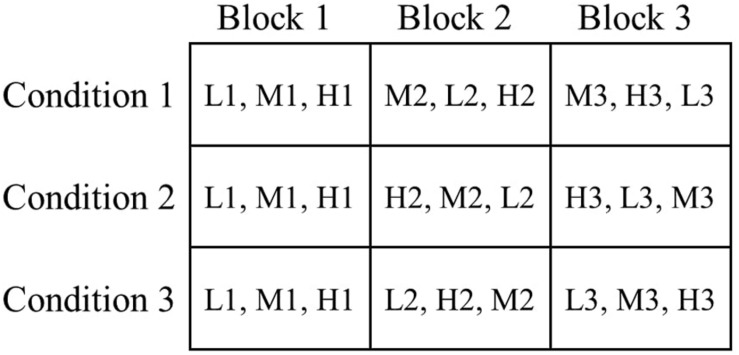
Experimental sequence design of the study. The nine study items were distributed across three different blocks. Each block contains one item with low visual load (L), one item with medium visual load (M), and one item with high visual load (H). The numbers after the letters represent the incidental-ID of the respective item (see Figure 1). All participants passed the three blocks in the same order. Participants were pseudo-randomly assigned to one of three conditions, meaning that the random number generator targeted an equal distribution of participants across the conditions. The condition determined the order of items within each block. For block 1, the order of items was the same for all conditions (from easiest to most difficult). For blocks 2 and 3 the order of items was varied. Within blocks 2 and 3, every magnitude of visual load appeared once in every order-position (from 1 to 3) across the conditions.

After these introductions, the remaining nine figumem items were successively presented to each participant. To control for sequence effects, the order of these nine items was varied across examinees. Instead of completely randomizing the order of items, their difficulty should be controlled for in the sequences. Presenting several difficult items at the beginning, for example, can have detrimental effects on participant motivation and performance. We therefore decided to restrict the possible presentation orders, systematically assign participants to groups defined by presentation order, and empirically check sequence effects via group comparisons. We oriented the design for this on Latin squares (e.g., Jacobson and Matthews, 1996): One item of each family was assigned to a block, resulting in three different blocks with three different items each. All participants went through all the blocks in the same order. However, we varied the order in which the three items of varying difficulty of a single block were presented between participants. At the beginning of the survey, participants were assigned to one of three conditions. In all conditions, the three items of the first block were presented in order of increasing difficulty to create a warm-up phase for all participants. In blocks 2 and 3, the order of item difficulties was rotated so that in each block, each item difficulty level appeared once at every position (first, second, third) across the conditions (see Figure 3). The algorithm of the survey tool aimed at a randomized assignment of participants to the conditions while simultaneously maintaining roughly equal sizes of condition groups. Since the algorithm could not consider the cancelation of participation of some examinees and could (obviously) not predict the exclusion of some participants (see the section “Sample”), group sizes were not equal in the end but still fairly balanced. 63 participants were in condition group 1, 76 in condition group 2, and 69 in condition group 3.

At the end of the survey, participants were asked for their socio-demographic information. Psychology students were informed about the procedure they had to undertake to gain their course credit.

### Analytic Strategy

We first fitted the general RPCM containing the items themselves as predictors (Eq. 3) as a GLMM to the data. Model fit was evaluated by several measures.

Firstly, we compared the model to a “person-only” model via a likelihood-ratio test. In this person-only model, person parameters are modeled as a random intercept, but item easiness parameters are not included, thereby assuming equal difficulty for every item. Secondly, we computed the dispersion index. If the expectation and variance of the outcome are identical (equidispersion), as the Poisson distribution requires (see Baghaei and Doebler, 2018), the dispersion index equals 1. Overdispersion (variance is greater than the expectation) is given by values greater than 1. Underdispersion (variance is lower than the expectation) is given by values smaller than 1. While overdispersion in the data leads to underestimation of standard errors, underdispersion leads to overestimation thereof. Hence, underdispersion may be considered less of a problem because it generally makes the scientific approach more conservative. Thirdly, we examined the Pearson residual plot for the complete model and Pearson residual boxplots for the single items. Additionally, the covariate adjusted frequency plot (Holling et al., 2013) was employed for a graphical model check. Fourthly, we checked item fit by χ^2^-tests as suggested by Baghaei and Doebler (2018). Here, we ordered participants by their total scores and placed them into five groups corresponding to the 0.20, 0.40, 0.60, 0.80, and 1.00 quantiles of total score distribution. For every item, a test statistic was computed that is based on a comparison of expected and observed item scores across the different groups. If the model holds, then this test statistic asymptotically follows a χ^2^-distribution with the degrees of freedom equal to the number of groups (= 5 in this case). A non-significant test result suggests good fit of the item. Fifthly, a differential item functioning analysis was utilized to compare the three different condition groups (that encountered the items in different orders). For this approach, the RPCM is enhanced by including group variables and their interactions with the items. Non-significant interaction terms signal identical item difficulty across groups. If that can be assumed for the complete test, another extended RPCM is estimated and interpreted that contains the group variables without interactions. An absence of significant group effects in this model suggests equal mean ability of the persons across the condition groups. The RPCM and these two extended versions of it were compared via likelihood-ratio tests (see Baghaei and Doebler, 2018, for details). Lastly, the reliability of the test grounded in the RPCM for the estimation of the person parameters was examined. In the RPCM, test reliability increases for increasing person parameter values. It is defined as

$$s_{\tilde{\theta }}^{2}/\left(s_{\tilde{\theta }}^{2}+s_{v}^{2}\right)$$

where $s_{\tilde{\theta }}^{2}$ gives the estimated variance of the person parameters (on log-level) and $s_{v}^{2}$ represents the squared standard error for the ability parameter for person *v* (Baghaei and Doebler, 2018).

We then estimated a new model in which the item parameters of the RPCM are replaced with the radical levels of the item model (Eq. 5). We refer to this model as the RPCM-r. A likelihood-ratio test between the RPCM and RPCM-r revealed whether implementing the assumption that item parameters can be completely explained by the radical decreased model fit significantly. Based on the RPCM, we investigated the differences in expected item score between items from the same family with different incidental-IDs.

The correlation of the item parameters from the RPCM and predicted item parameters from the RPCM-r was investigated. We examined the correlation between the person parameters of the RPCM and the person parameters of the RPCM-r, as well. Finally, we judged overall fit of the RPCM-r via graphical model checks and investigated the reliability of its produced person parameter estimates.

For further information on the procedures, we refer to Baghaei and Doebler (2018) and Holling et al. (2013). All models were estimated with the “lme4” software package (Bates et al., 2015). A data set and an R-script containing all analyses are available as online Supplementary Material.

## Results

Table 1 shows descriptive statistics of all test items. Mean scores decreased with increasing visual load as determined by the radical, and so did item score standard deviations. The easiest item (radical level 1, incidental-ID 3) had a mean score of 11.46 (SD = 4.18) correctly remembered associations. The most difficult item (radical level 3, incidental-ID 3) had a mean score of 7.12 correctly remembered associations (SD = 2.90). No part–whole corrected discrimination parameter of any item fell below 0.40. Cronbach’s alpha for the complete test was α = 0.85 (95% confidence interval = [0.82; 0.88]). Table 2 displays the Pearson correlations of the items.

**TABLE 1:** Descriptive statistics and χ^2^-tests of item fit.

Item		Statistic							

R	I	Median	Mean	SD	Minimum	Maximum	Discrimination	χ ^2^ (*df* = 5)	*p*-value
1	1	11.00	10.70	3.58	2	18	0.48	4.23	0.517
1	2	11.00	11.09	3.82	1	19	0.65	6.32	0.277
1	3	11.50	11.46	4.18	2	20	0.64	4.66	0.458
2	1	9.00	9.48	3.26	2	19	0.58	1.09	0.955
2	2	10.00	9.80	3.60	2	20	0.67	1.08	0.956
2	3	8.00	8.66	3.72	1	18	0.66	0.83	0.975
3	1	7.00	7.22	3.02	2	18	0.49	2.31	0.804
3	2	7.00	7.58	2.86	0	19	0.53	6.33	0.276
3	3	7.00	7.12	2.90	1	17	0.49	4.37	0.498

**N* = 208; R = radical level; I = incidental-ID; SD = standard deviation; discrimination = part–whole corrected discrimination parameter. Radical determined visual load (1, low; 2, medium; 3, high).*

**TABLE 2:** Pearson correlations of study items.

	R1 I1	R1 I2	R1 I3	R2 I1	R2 I2	R2 I3	R3 I1	R3 I2
R1 I2	0.400***	–	–	–	–	–	–	–
R1 I3	0.396***	0.608***	–	–	–	–	–	–
R2 I1	0.370***	0.427***	0.378***	–	–	–	–	–
R2 I2	0.456***	0.519***	0.527***	0.447***	–	–	–	–
R2 I3	0.346***	0.496***	0.536***	0.442***	0.515***	–	–	–
R3 I1	0.243***	0.325***	0.296***	0.409***	0.381***	0.351***	–	–
R3 I2	0.214**	0.388***	0.352***	0.376***	0.355***	0.482***	0.319***	–
R3 I3	0.196**	0.312***	0.311***	0.337***	0.359***	0.379***	0.432***	0.454***

*N* = 208. **p < 0.01, ***p < 0.001.

### Rasch Poisson Counts Model With Items as Predictors

The RPCM fitted the data significantly better as a person-only model that omits the item parameters [χ^2^(8) = 512.93, *p* < 0.001]. The standard deviation of the ability parameters from the RPCM was estimated to be 0.229 (95% confidence interval = [0.203; 0.258]). The third column of Table 3 displays item parameters of the RPCM on the counts level. The expected item score decreased with increasing visual load, yet parameters also differed within item families. The dispersion index was φ = 0.72. Thus, data were underdispersed. Underdispersion should be considered for diagnostic purposes, as it also underestimates the reliability of person parameter estimates. We investigate the problem of underdispersion for the current case at the end of the “Results” section.

**TABLE 3:** Item easiness parameters (counts level) for various models (95% confidence interval bounds in rectangular brackets).

Item		Model					

R	I	RPCM	CMP-gd	CMP-sd	RPCM-r	CMP-r-gd	CMP-r-sd
1	1	10.43 [9.90; 10.98]	10.41 [9.91; 10.94]	10.41 [9.90; 10.96]	–	–	–
1	2	10.81 [10.26; 11.37]	10.79 [10.28; 11.33]	10.79 [10.29; 11.32]	10.80 [10.38; 11.23]	10.79 [10.38; 11.21]	10.79 [10.38; 11.21]
1	3	11.16 [10.60; 11.74]	11.15 [10.62; 11.70]	11.15 [10.61; 11.72]	–	–	–
2	1	9.24 [8.74; 9.75]	9.23 [8.77; 9.71]	9.23 [8.79; 9.69]	–	–	–
2	2	9.55 [9.05; 10.07]	9.54 [9.07; 10.03]	9.54 [9.08; 10.02]	9.08 [8.71; 9.45]	9.07 [8.71; 9.43]	9.07 [8.72; 9.43]
2	3	8.44 [7.98; 8.92]	8.43 [8.00; 8.89]	8.43 [7.99; 8.90]	–	–	–
3	1	7.04 [6.63; 7.46]	7.03 [6.65; 7.43]	7.03 [6.64; 7.44]	–	–	–
3	2	7.38 [6.96; 7.82]	7.37 [6.98; 7.79]	7.37 [6.99; 7.77]	7.12 [6.82; 7.43]	7.11 [6.82; 7.41]	7.11 [6.82; 7.41]
3	3	6.93 [6.53; 7.36]	6.92 [6.55; 7.32]	6.92 [6.54; 7.33]	–	–	–

**N* = 208. RPCM = Rasch Poisson counts model with items as predictors; CMP-gd = Conway–Maxwell–Poisson model with items as predictors and a global dispersion parameter; CMP-sd = Conway–Maxwell–Poisson model with items as predictors and item-specific dispersion parameters; RPCM-r = Rasch Poisson counts model with the radical as a predictor; CMP-r-gd = Conway–Maxwell–Poisson model with the radical as a predictor and a global dispersion parameter; CMP-r-sd = Conway–Maxwell–Poisson model with the radical as a predictor and radical level–specific dispersion parameters. Parameters reflect expected item scores given average ability.*

**FIGURE 4:**
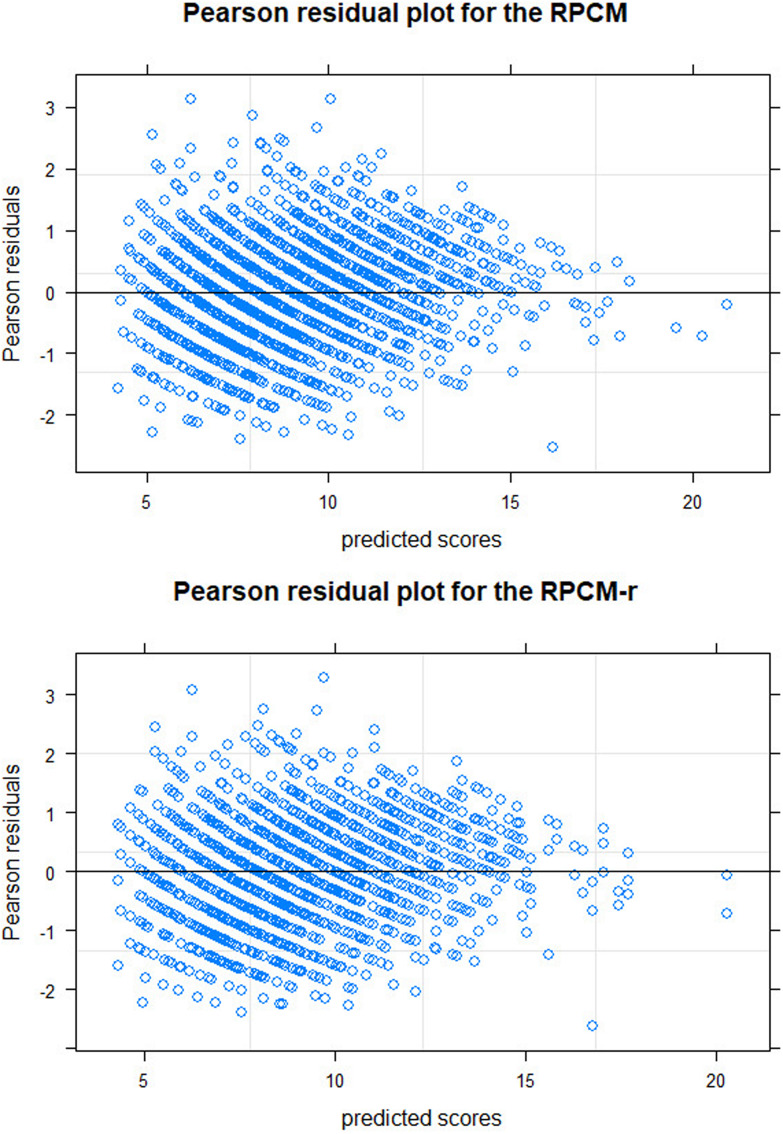
Pearson residual plots for the RPCM (Rasch Poisson counts model with items as predictors; upper graph) and for the RPCM-r (Rasch Poisson counts model with the radical as a predictor; lower graph).

The top graph of Figure 4 shows the Pearson residual plot. Residuals roughly ranged from -2 to 2 with a few outliers. They are fairly symmetrical against the zero-axis. The residual variance was reduced for higher predicted scores, again displaying the underdispersion in the data. Overall, the plot displays a satisfactory model fit.

**FIGURE 5:**
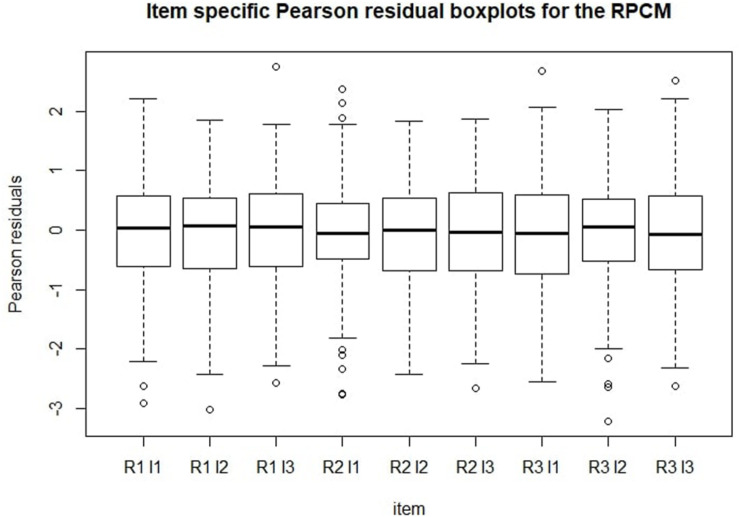
Item-specific Pearson residuals boxplots for the RPCM. R = radical level; I = incidental-ID.

Figure 5 shows Pearson residual boxplots for item-specific predicted scores. Generally, the same conclusions can be derived from this graph as from Figure 4. Mean residuals were around zero for every item. As expected, most residuals settled between -2 and 2.

**FIGURE 6:**
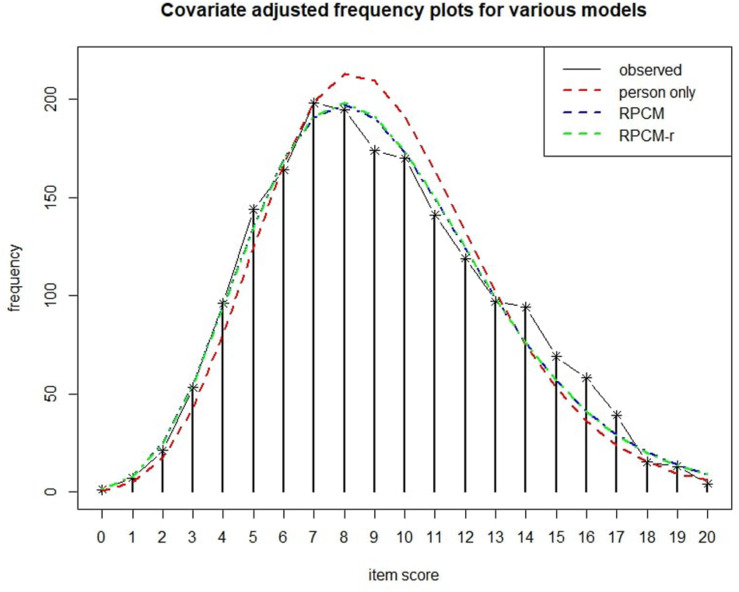
Covariate adjusted frequency plot (Holling et al., 2013) for various models. The person-only model contains only person parameters as a random intercept for score prediction.

The blue dashed line in Figure 6 shows the expected frequency of item scores as predicted by the RPCM. As can be seen, the line approaches the observed scores closely.

The last two columns of Table 1 deliver the results of the item-based χ^2^-tests. All *p*-values were above 0.20, suggesting satisfactory fit for every item.

The differential item functioning approach revealed a significant difference for the difficulty of the item with radical level 3 and incidental-ID 1 between the first and the second group (β = −0.17, *z* = −2.06, *p* = 0.039). However, the multiple-testing issue due to several groups and items must be considered in this approach (see Baghaei and Doebler, 2018). Some significant differences were expected to occur by chance. No other interaction effect was significant (range of *p*-values = 0.058–0.919). A likelihood-ratio test revealed no significant decline in model fit when interactions between items and group variables were restricted to zero and only additive effects of the group variables were maintained in the model [χ^2^(16) = 16.93, *p* = 0.390]. When the group variables were added into the RPCM without any interaction terms, they had no significant effect on the item score (β_*2*_ = −0.02, *z* = −0.58, *p* = 0.564, 95% confidence interval = [−0.11; 0.06]; β_*3*_ = −0.03, *z* = −0.59, *p* = 0.559, 95% confidence interval = [−0.11; 0.06]). A likelihood-ratio test revealed no significant difference in fit between this model and the RPCM [χ^2^(2) = 0.44, *p* = 0.803], suggesting equal mean ability across groups.

**FIGURE 7:**
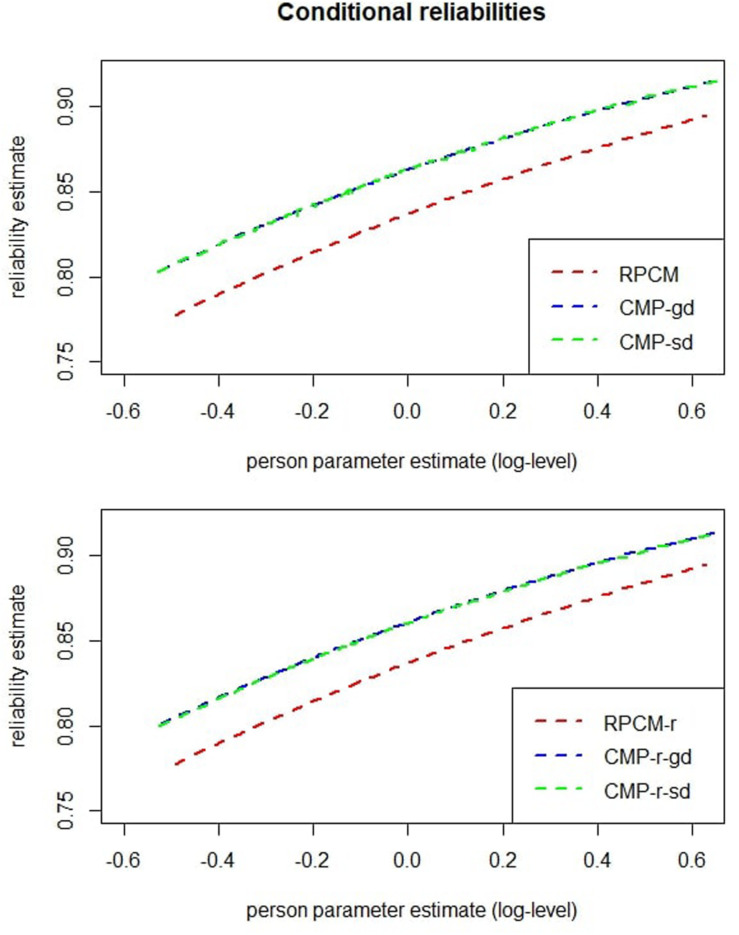
Conditional reliabilities of person parameters for models with items as predictors (upper graph) and for models with the radical as a predictor (lower graph). CMP-gd = Conway–Maxwell–Poisson model with items as predictors and a global dispersion parameter; CMP-sd = Conway–Maxwell–Poisson model with items as predictors and item-specific dispersion parameters; CMP-r-gd = Conway–Maxwell–Poisson model with the radical as a predictor and a global dispersion parameter; CMP-r-sd = Conway–Maxwell–Poisson model with the radical as a predictor and radical level–specific dispersion parameters.

The red dashed line in the top graph of Figure 7 plots the estimated reliability of ability estimation against the person parameters as estimated within the RPCM. The smallest conditional reliability was α = 0.78. As already mentioned, this reflects an underestimation due to underdispersion, and a better estimate will be given at the end of the “Results” section.

During the revision of this article, one of the reviewers pointed out that a motivational bias might have impacted the results since the non-psychology students did not receive any reimbursement for participation and should therefore do worse on the tasks. We refitted the RPCM to the data and included a binary variable that indicated whether a participant was a psychology student or not. The model predicted the psychology students to remember 2.24% less figural associations than the non-psychology students, but this difference was not significant (*p* = 0.528). Hence, no evidence for this type of motivational bias was present in the data.

### Influence of the Radical and the Incidentals

The person parameter standard deviation and its 95% confidence interval bounds within the RPCM-r were identical to the respective statistics from the RPCM when rounded to the third decimal. The sixth column in Table 3 presents estimates and 95% confidence intervals of item easiness for every level of the radical variable on the counts level. The non-overlapping confidence intervals reveal that the expected item score decreased significantly with increased visual load. In other words, items with higher visual load were significantly more difficult. The dispersion index for the model was φ = 0.74.

A likelihood-ratio test between the RPCM and the RPCM-r revealed a significant decline in model fit when item parameters were assumed to be fully explained by the radical [χ^2^(6) = 24.21, *p* < 0.001]. Table 4 contains differences in expected item scores between items with the same radical level (i.e., from the same family) but with different incidental-IDs. Incidentals did not impact the expected score on items with high visual load. Within the family of low visual load, two items had a significantly different difficulty. Within the family of medium visual load, one item difficulty was significantly different from the others. The practical implications will be evaluated in the section “Discussion.”

**TABLE 4:** Differences in expected item scores between items with different incidental-IDs for each level of the test radical.

Difference	Radical level = 1	Radical level = 2	Radical level = 3
ID 2 - ID 1	+3.57% (+0.38)	+3.34% (+0.31)	+4.81% (+0.35)
ID 3 - ID 1	+6.82% (+0.74)*	−9.01% (−0.80)**	−1.48% (−0.10)
ID 3 - ID 2	+3.24% (+0.36)	−12.36% (−1.11)***	−6.29% (−0.45)

*N* = 208. Radical level determined visual load (1, low; 2, medium; 3, high). Numbers in parentheses give the expected difference in item score for a person with average cognitive ability. Significant differences are printed in bold. *p < 0.05, **p < 0.01, ***p < 0.001.

The correlation between item parameter estimates of the RPCM and predicted item parameters of the RPCM-r was *r* = 0.977 (*p* < 0.001). Hence, the radical explained 95.41% of the variance in item difficulty in the general RPCM (adjusted *R*^2^ = 0.939). The correlation between the person parameters of the two models was *r* = 1.

The bottom graph of Figure 4 displays the residual plot for the RPCM-r. It is very similar to the residual plot for the RPCM and suggests satisfying model fit. The green line in Figure 6 shows the expected frequency of item scores based on the RPCM-r. It is nearly identical to the predictions of the RPCM indicating no decline in the accuracy of the expected scores when the radical is used as a predictor of item easiness instead of estimating item parameters directly. The red line in Figure 6 shows the fit of the person-only model in which items were not assumed to differ in difficulty. As our cognitive model is rather simple and implies a uni-dimensionality of visual load, this model fitted the data rather well, too. However, it substantially underestimated the frequency of lower item scores and overestimated the frequency of medium item scores by not taking the difficulty of items into account.

The red dashed line in the bottom graph of Figure 7 displays reliability estimates of person parameters for the RPCM-r. A comparison with the top graph of Figure 7 reveals no cutbacks on reliability when the RPCM-r was used instead of the RPCM.

### *Post hoc* Analyses for the Underdispersion

Underdispersion violates a core assumption of the RPCM. However, even for underdispersed data, the RPCM (or the RPCM-r in the current case) may still be a useful and valid approximation of the true model with regard to the estimation of person and item parameters. It depends on the robustness of parameter estimates against the violation of equidispersion.

Brooks et al. (2017) published the R package “glmmTMB,” which allows the estimation of Conway–Maxwell–Poisson (CMP) counts models. These models can be considered generalized Poisson counts models that relax the assumption of equidispersion by either estimating a global dispersion parameter or several item (or, more generally, predictor) specific dispersion parameters (Forthmann et al., 2019a, b). We modified the RPCM into a CMP model containing a global dispersion parameter (referred to as the CMP-gd) and into a CMP model with item-specific dispersion parameters (referred to as the CMP-sd). Similarly, we modified the RPCM-r into a CMP model containing a global dispersion parameter (CMP-r-gd) and into a model containing radical level–specific dispersion parameters (CMP-r-sd). Table 5 shows comparisons of nested models with likelihood-ratio tests and Akaike information criteria (AICs) and Bayesian information criteria (BICs).

**TABLE 5:** Model comparisons between Rasch Poisson models and Conway–Maxwell–Poisson models.

Model	Δχ ^2^(*df*)	*p*-value	AIC	BIC
RPCM	–	–	9,422.10	9,477.50
CMP-gd	34.18 (1)	<0.001	9,389.90	9,450.80
CMP-sd	8.85 (8)	0.355	9,397.10	9,502.30
RPCM-r	–	–	9,434.30	9,456.50
CMP-r-gd	28.88 (1)	<0.001	9,407.50	9,435.10
CMP-r-sd	0.77 (2)	0.680	9,410.70	9,449.40

**N* = 208. AIC = Akaike information criterion; BIC = Bayesian information criterion.*

As can be seen, for both the RPCM and the RPCM-r, the modification with the inclusion of a global dispersion parameter improved model fit significantly. This is to be expected when underdispersion was found in a Poisson model. Modeling item or radical level–specific dispersion parameters, however, had no significant incremental effect on model fit. The fourth and fifth columns of Table 3 reveal that item parameter estimates within the CMP-gd and CMP-sd are almost identical to the ones derived from the RPCM. The confidence interval bandwidths become smaller. The seventh and eighth column of Table 3 display radical parameters for the CMP-r-gd and the CMP-r-sd. Again, only marginal and non-substantial changes in comparison to the RPCM-r were observed.

Person parameters from the RPCM correlated with *r* = 1 with the person parameters from the CMP-gd and with *r* = 0.999 with the person parameters from the CMP-sd, respectively. Person parameters from the RPCM-r correlated with *r* = 1 with the person parameters from the CMP-r-gd and with the person parameters from the CMP-r-sd. Taken together, these results justify staying with the more parsimonious Rasch Poisson models, as the robustness of person and item parameters against the violation of equidispersion is demonstrated for this case.

The upper graph of Figure 7 displays conditional reliabilities of the RPCM, the CMP-gd, and the CMP-sd; the lower graph of Figure 7 displays conditional reliabilities of the RPCM-r, the CMP-r-gd, and the CMP-r-sd. As can be seen, reliabilities were substantially underestimated within the RPCM and the RPCM-r due to underdispersion. For the CMP-gd and the CMP-r-gd, the smallest reliability was α = 0.80.

## Discussion

The current work introduced figumem, a figural memory test based on AIG readily available for researchers and practitioners in R. The cognitive model implemented in the item generator relates to the phenomenon of visual information load. Three different item difficulties determined by the magnitude of visual load can be differentiated. Psychometrically similar items for each magnitude of visual load can be created by randomly determining the concrete figural associations that are to be memorized and their presentation order. An empirical study mostly confirmed the qualities of the item generator. Various analyses with different modifications of the RPCM justified specific objective comparisons via raw scores. The item generator radical (visual load) determined the item difficulty. Variance in item difficulty due to the item generator incidentals was small in comparison yet not negligible (see below). Figumem holds the potential for an efficient, reliable, and repeatable assessment of figural memory in various non-clinical and clinical populations. It should be stressed that the test so far is not normed and should only be used for research purposes. Nevertheless, the test’s potential for diagnostic use should be considered. In the following, we discuss the implications of certain results, practical implications for the use of figumem, and limitations of the pilot study.

### Implications of the Results

Since some items from the same item family with different incidentals differed significantly in difficulty, true psychometrical equivalence of isomorphs cannot be assumed. However, the variation in item difficulty within an item family can be dealt with effectively in practice. For example, in the group of items with low visual load, the item with incidental-ID 3 was significantly easier than the item with incidental-ID 1. The RPCM predicts an increase of 6.82% of the score on the item with incidental-ID 1 for the score on the item with incidental-ID 3 (see Table 4). For the average person, this equals 0.74 additional expected correctly remembered associations that can be traced back to variation in item surface features. This is actually not much given that the average person was able to memorize around 10–11 figure associations of items with low visual load (see Table 3). It is around a fifth of the standard deviation for the item with radical level 1 and incidental-ID 1 (see Table 1). When using items with low visual load but different incidentals for a longitudinal assessment, it is then recommended to take a conservative stance in the diagnostic procedure and interpret a change in item score of plus or minus 1 with caution. Note that in a clinical context, when the STM capacity of a patient is severely impaired, lower item scores are expected, and variation of the item score due to item incidentals is reduced accordingly. In these cases, the variation can be so small that the precision of the instrument is no longer able to capture it. It follows that a significant increase in test score over time can then be validly traced back to changes within the testee, and the use of several items from the same item family is valuable for the obviation of retest effects.

Regarding items with medium visual load, the variation within the item family is more severe. For example, the RPCM predicted a decrease of 12.36% of the score on the item with incidental-ID 2 for the score on the item with incidental-ID 3. Thus, the latter mentioned item was significantly more difficult. This might still not be substantial, especially in clinical scenarios, but items with medium visual load should be used with more caution. For a mere measurement of visual STM, it is recommended to rely on the use of low-visual-load items. The RPCM has the convenient property that the easiest items can effectively differentiate between persons from the complete ability spectrum as long as floor and ceiling effects are not present (Holling and Schwabe, 2016). While three participants reached the maximum score of 20 for the item with radical level 1 and incidental-ID 3, It is important to keep in mind that our sample consisted mainly of students and academics, who are expected to be well above average on the ability spectrum.

The item families with more visual load should be considered with regard to construct validity, as they present potential for further studies on memory. In this study, visual information load predicted most of the variance in item easiness (95.41%). This can be seen as a strong confirmation of our cognitive model (Arendasy et al., 2008). Moreover, our results thereby fall in line with the findings of Alvarez and Cavanagh (2004), although we used different stimulus material, different measurement techniques, and an overall different experimental design. While this might also hint toward high construct validity, future studies are required to explore the correlations of test performances on figumem items and already established testing procedures and experimental assessment techniques. In that context, a broader framework that extends STM to the multiple facets of WM should be established. While STM can be differentiated from WM by the given task (memorization vs. manipulation; see above), it is not obvious that the memorization of more complex (i.e., more visually loaded) stimulus material does not occupy the same cognitive processes such as attention, imagery, and executive functions that are debated to be central to WM (Kosslyn, 2005; Baddeley et al., 2011; Logie, 2011; Baddeley, 2012; Slana Ozimič and Repovš, 2020). For example, in order to memorize figural associations from figumem items with medium and high visual load, it might be necessary to not only rehearse the associations in an *inner scribe* (the rehearsal mechanism of the visuospatial sketchpad; see Baddeley, 2012, p. 13) but also actively compare two similar frames with regard to the emblems they contain. From this perspective, one would expect higher correlations between more demanding WM tasks and visually more loaded figumem items compared to visually less loaded figumem items.

### Practical Implications and Additional Features of Figumem

Within every item created by figumem, all 20 emblems and all 20 frames available for the respective item family are used. This raises the question of how many items that substantially differ in their presentation features can be truly created. Participants of our study were mainly recruited among academics, and the field test revealed restricted variance of test outcomes not to be an issue (Table 1 and Figure 6). When using figumem for the diagnosis of people from the average of the ability spectrum or from clinical populations, the amount of presented emblem–frame units can be reduced, which in turn increases the amount of concrete figural associations that can appear in an item. This feature is already implemented in figumem. Alternatively, more figures can be created to enhance the item generator. This would demand a repeated empirical evaluation of the item model.

Although the implemented cognitive model of figumem relates to visual STM, the test should not be regarded as a distinct measure of STM alone. Various models of memory functions in general coexist and differ in their degrees of psychometrical and neuropsychological grounding. Different working definitions for memory are used in order to achieve specific aims, like the explanation of certain memory phenomena or the precise diagnosis of a neurological condition (Murdock, 1962; Glanzer and Cunitz, 1966; Baddeley, 1992; Frankland and Bontempi, 2005; Purves et al., 2013; Goldstein, 2014). The use of specific test material can be adjusted in accordance with the working definition. For a simple example, the Rey–Osterrieth complex figure test (Rey, 1941) is often used to assess visual STM, as the testees must reproduce (i.e., draw) a figure in an open response format immediately after visually inspecting it. Then, after some time has elapsed and the testees have worked on different tasks, they must draw it again from memory. This is then considered a performance of visual long-term memory retrieval because the different tasks during the delay period should have occupied STM and WM. Figumem can be used in the same way. An open response format is implemented in the software. It can be used to produce a matrix of emblem–frame units. Templates only containing the emblems can be generated alongside. The testee’s task is to draw the frames around the emblems of this template.

With respect to different models of memory, extensions of the cognitive model of the item generator should be considered. Unlike fluid reasoning, in which inductive and deductive cognitive processes are rather precisely defined (Cattell, 1987; McGrew, 2009), the scientific definition of basic STM relates to mere capacity, not processes. As previously discussed, the concept of WM goes beyond that of STM because elements must be not only remembered but manipulated as well. This implies specific forms of conscious cognitive processing. In that context, the actual differences between WM and fluid reasoning are debated (e.g., Kyllonen and Christal, 1990; Kane et al., 2004). Extending figumem for the measurement of WM is possible as item formats in which testees must manipulate the placing of missing lines of the frames and orientation of the emblems themselves are feasible. The number of radicals can be increased to contain, for example, colors for the emblems and dashed and dotted presentations of frame lines. This would further require an extension of the cognitive model and its statistical representation (see Freund et al., 2008).

### Limitations

We drew a sample of nine figumem items for the empirical investigation of the item generator. This is obviously a very small sample size, and our results should be generalized with caution. Replications of the test of figumem should include a larger number of incidental realizations for every radical level. This can be problematic from a practical viewpoint, as visual memory tests are perceived as rather cumbersome by participants. An expedient alternative in study design is to randomly sample the incidentals for every individual participant instead of using a fixed set of items across all examinees. This requires the production of test items “on the fly.” If one aims at conducting the inquiry online, then this approach is bound to advanced survey tools that are not readily available to any researcher. However, such a study design is especially useful for better estimating the variance of item difficulty within an item family. Alternatively, the level of analysis can be altered when the influence of incidentals on item difficulty is investigated. Instead of looking at the sum of correctly remembered associations for every item as the smallest bit of information, as it is done in the RPCM, one can also model the probability for correctly remembering every specific emblem–frame association directly. The advantage of this approach is that specific emblem–frame associations that are hypothesized to be easily remembered due to the employment of other cognitive processes or test-specific strategies can be assessed directly. However, it is not a straightforward task to generate such hypotheses in an efficient way. The authors experienced this in a post-study briefing. For example, an argument was made that the emblem that looks like a plus sign should not be presented inside the square-shaped frame, because such a constellation bears resemblance to the Swiss flag and will be remembered with this mnemonic instead of visual memory. This hypothesis, however, can only be potentially confirmed for testees with a certain minimal knowledge about national flags. For a rather specific subset of participants, it might be more crucial to avoid combining the emblem with the four arrows and the square-shaped frame because this reminds them of a movable platform in a specific action-adventure video game. The important point is that, while mnemonic strategies for some figure combinations might be more common than others, they can probably not be fully controlled for. One should bear in mind that, in an AIG context, the practical implications of observed variance in difficulty within an item family are often more meaningful than its statistical significance (see section “Implications of the Results”).

## Conclusion

Figumem is a promising tool for the measurement of figural memory capacity and readily available in R. It is based on a cognitive model related to the visual information load phenomenon. Due to the AIG, it theoretically allows for the production of a very large number of items with similar psychometric properties. An empirical study displayed the fit of the test to the RPCM and the item generator’s capability for the creation of psychometrically similar items within an item family defined by visual load. Extensions of the visual material are feasible. Future research with larger samples of automatically generated items is needed to further generalize the qualities of the item generator with regard to construct validity, different populations, different item formats, and the assessment of long-term memory.

## Data Availability Statement

The data sets (“Data.rda”) for this study can be found in the Supplementary Material.

## Ethics Statement

Ethical review and approval was not required for the study on human participants in accordance with the local legislation and institutional requirements. Written informed consent from the participants’ legal guardian/next of kin was not required to participate in this study in accordance with the national legislation and the institutional requirements.

## Author Contributions

DJ, LB, and HH: conceptualization, methodology, and writing – review and editing. DJ: software, formal analysis, data curation, writing – original draft preparation, and visualization. DJ and LB: validation, investigation, and resources.

## Conflict of Interest

The authors declare that the research was conducted in the absence of any commercial or financial relationships that could be construed as a potential conflict of interest.

## Footnotes

1 Due to the random sampling, a specific emblem–frame combination can occur across different items by chance, and the position of a specific emblem or a specific frame can remain unchanged across different items by chance.
