# Supplementary material for: Introducing a Computerized Figural Memory Test Based on Automatic Item Generation: An Analysis With the Rasch Poisson Counts Model
Source: Front Psychol. 2020 Jun 10;11:945. doi: 10.3389/fpsyg.2020.00945 (PMC7298330; doi:10.3389/fpsyg.2020.00945)
Supplement: Supplementary file 1 [file Data_Sheet_1.ZIP › supplementary_material/figumem.zip!/figumem/figumem_Manual.docx]

**FIGUMEM – USER’S MANUAL**


Correspondence: David Jendryczko (david.jendryczko@uni-konstanz.de)
 **Contents**

**Preface**	2
**Preparations**	3
**Explanation of the figumem_item-function**	4
*Example items*	6
**Explanation of the figumem_write-function**	8
*Examples*	8
**References**	11

**Preface**
This manual describes how to use the item generator *figumem* for the creation of figural memory items for cognitive ability assessments. It is strongly recommended to read the article *Introducing a computerized figural memory test based on automatic item generation: An analysis with the Rasch Poisson counts model* first, as it explains figumem and delivers the theoretical backgrounds on automatic item generation and the construct of figural memory.
The following files are provided:
(1) The R-script “run_me.R”. This script contains the required functions to generate and save figumem items.
(2) 120 PNG data files containing images of the frames and emblems of figumem. They have to be read into R to be further processed by the figumem item generator. There are 20 frames for items for the first difficulty level (“easy”) and 20 different frames for items for the second (“medium”) and the third (“hard”) difficulty level. There are 20 emblems for items for the first and second difficulty level. 20 more emblems are provided for the third difficultly level. Every emblem comes with a small and a large version in separate PNG files. This ensures a sharp presentation of the emblems across the different item formats. The names of the PNG files are chosen in a way that explains their content. For example, the first frame for the second and third difficulty level is given within the file “frame_medium_hard1.png” and the twentieth emblem for the first and the second difficulty level in a small format is given within the file “emblem_small_easy_medium20.png”.
The easiest way to read the PNG files into R is by saving them in the file that also contains “run_me.R” (which is the case in the provided ZIP file) and then running the script (for further details see “Preparations”). The use of figumem requires knowledge about the very basics of the functionality of R. Literature on this is given, for example, by Teetor (2011). If you wish to modify figumem for your specific research-schemes, feel free to alter the code in the script “run_me.R”. We also recommend the study of the magick software package (Ooms, 2018) for further insight into the possibilities of image-manipulation in R. For that purpose visit <https://cran.r-project.org/web/packages/magick/vignettes/intro.html>.

**Preparations**
To install the free software R on your computer visit
<https://www.r-project.org/>.
The use of RStudio is recommended. This is an add-on to R that graphically modifies its user-interface in a more comprehensible way. For the installation of RStudio R needs to be installed beforehand. To download the RStudio installer visit
<https://rstudio.com/products/rstudio/download/>.
The generation of figumem items requires the free R-software package magick (Ooms, 2018) additionally. The script ‘run_me.R’ takes care of the installation but requires an internet connection.
Set your R-working directory to the file in which the PNG data files containing the figumem emblems and frames and the script “run_me.R” are saved. In order to do so, run the following code in the R console

setwd("path")

whereas path needs to be replaced with the path to your directory. Alternatively, you can set your working directory in RStudio by clicking *Session 🡪 Set working directory 🡪 Choose directory*.
Run the script ‘run_me.R’. In order to do so, click somewhere into the script. Then, hold ‘Ctrl’ and press ‘A’. Finally, hold ‘Ctrl’ and press the return-button. After the script has run, you can use the functions figumem_item which creates figumem items and figumem_write which saves figumem items as PNG data files.

**Explanation of the figumem_item-function**
figumem_item creates figumem items. The function contains the following arguments:

figumem_item(objn = 20, mode = "classic", challenge = 1, seed = sample(1:999999999,1), ntrys = 100)

objn determines how many emblem-frame-associations shall be used. The default is 20 but it can be any natural number between one and twenty.
mode determines the item format. Currently, four formats are available. The default is "classic".
"classic" creates figumem item material for an easy digital implementation of a figural memory item into common online survey tools. One image is produced that contains the memorizing display, i.e. the emblems in their assigned frames arranged in a matrix. One image per emblem is produced that contains a large representation of the emblem. One image per emblem is produced that contains the target frame of the emblem that surrounds it in the memorizing display. Three images per emblem are produced that contain distractor frames for the emblem.
"classic_paper" reduces the amount of produced images. One image is produced that contains the memorizing display. One image is produced that contains a matrix in which the emblems are presented in a different order with four frames (one target, three distractors) arranged underneath each emblem.
"draw" creates figumem item material for an open response format that requires testees to draw the frames. One image is produced that contains the memorizing display. One image is produced that contains the emblems in the same order as before but without the frames.
"draw_random" is the same as "draw" but the order of the emblems for the second image (without the frames) is randomized.
challenge can be seen as the item generator radical and determines the item difficulty, which in turn is given by magnitude of visual load. 1 produces items of the easiest difficulty level (low visual load), 2 produces items of a medium difficulty level (medium visual load) and 3 produces items of the hardest difficulty level (high visual load). The default is 1.
seed sets the seed for the item generator. The assignment of frames to emblems, the order of the figural associations and the selection of distractors are determined randomly (these properties are incidentals). By specifying a seed number an item can be exactly reproduced in spite of that. The default is to use a random seed number.
ntrys determines how many tries the item generator shall take before a figumem item is successfully produced or before the generation process is cancelled. This is only relevant for the modes "classic" and "classic_paper" because the algorithm that randomly assigns distractors to emblems while simultaneously ensuring that every frame is used equally often does not always converge. The default is 100. If convergence does not occur within the specified number of tries, a warning message will be printed in the console that explains that a certain distractor could not be decided for and that a new attempt is required. It is recommended to always produce items using a fixed seed number. If the algorithm does not converge, the seed number can be altered and documented and a new attempt can be made.
*Example items*	To create an example figumem item in "classic"-format use the following code:

eg1 <- figumem_item(objn = 20, mode = "classic", challenge = 1, seed = 1, ntrys = 100)

Since in this case all function arguments except for seed are set to their defaults, the code can be written in an abbreviated form and will produce the same item:

eg1 <- figumem_item(seed = 1)

It creates the object eg1 that contains all the produced images. The images can be assessed. In order to examine the memorizing display use:

eg1[[1]]

In order to examine the first emblem use:

eg1[[2]][[1]][1]

In order to examine the second emblem use:

eg1[[2]][[2]][1]

In order to examine any of the third to the twentieth emblem replace the 2 in the middle set of brackets of the last code-line by the respective number. In order to examine the target frame, the first distractor, the second distractor and the third distractor of the first emblem in that order run the following lines successively:

eg1[[2]][[1]][2]
eg1[[2]][[1]][3]
eg1[[2]][[1]][4]
eg1[[2]][[1]][5]

The target frame and the distractors for the second to the twentieth emblem can be assessed by replacing the 1 in the middle sets of brackets of the last four code-lines with the respective number.

To create the same item in "classic_paper"-format use the following code:

eg2 <- figumem_item(mode = "classic_paper", seed = 1)

It creates the object eg2 that contains all the produced images. In order to examine the memorizing display use:

eg2[[1]]

In order to examine the answering display, in which the testee has to mark the correct options, use:

eg2[[2]]

To create the same example figumem item in "draw"-format use the following code:

eg3 <- figumem_item(mode = "draw", seed = 1)

It creates the object eg3 that contains all the produced images. In order to examine the memorizing display use:

eg3[[1]]

In order to examine the answering display, in which the testee has to draw the frames around the emblems, use:

eg3[[2]]

To create the same item in "draw_random"-format use the following code:

eg4 <- figumem_item(mode = "draw_random", seed = 1)

It creates the object eg4 that contains all the produced images. In order to examine the memorizing display use:

eg4[[1]]

In order to examine the answering display, in which the testee has to draw the frames around the emblems (that are now presented in a different order), use:

eg4[[2]]

**Explanation of the figumem_write-function**
figumem_write saves figumem items as PNG-files. The function contains the following arguments:

figumem_write(item, name = "figural_memory_item")

item specifies which item shall be saved in PNG format. This argument has no default.
name specifies the general name for the PNG data files. The default is “figural_memory_item”.
The PNG files will be saved in the current working directory. The working directory can be changed before the function is used (see “Preparations”).

*Examples*
In order to save the figumem item eg1 use:

figumem_write(item = eg1)

The function recognizes that eg1 is a figumem item in "classic"-mode. It will create several PNG data files in the current working directory. All of the file names will start with “figural_memory_item”. One file will be named “figural_memory_item_display.png”. It will contain the memorizing display, i.e. the image retrieved from eg1[[1]].
Twenty files will be named “figural_memory_item_stimulus_x_emblem.png”, whereas “x” is to be replaced with a number from 1 to 20, respectively. They will contain the emblems.
Twenty files will be named “figural_memory_item_stimulus_x_target.png”, whereas “x” is to be replaced with a number from 1 to 20, respectively. They will contain the target frames for the respective emblems.
Sixty files will be named “figural_memory_item_stimulus_x_distractor_w.png”, whereas “x” is to be replaced with a number from 1 to 20, respectively and “w” is to be replaced with a number from 1 to 3 for every given “x”, respectively. They will contain the distractors for the respective emblem given by “x”.
In order to save the figumem item eg2 use:

figumem_write(item = eg2, name = "figumem_paper_item")

By altering the expression for the name argument of the function one can assure not to overwrite previously saved items. The function recognizes that eg2 is a figumem item in "classic_paper"-mode. It will create two PNG data files in the current working directory. Both file names will start with “figumem_paper_item”. One file will be named “figumem_ paper_item_display.png”. It will contain the memorizing display, i.e. the image retrieved from eg2[[1]]. One file will be named “figumem_ paper_item_choices.png”. It will contain the display in which the testee has to mark the correct options, i.e. the image retrieved from eg2[[2]].
The function will also recognize items of the remaining formats ("draw" and "draw_random"). In these cases, two files – one file with the postfix “_display.png” and one file with the postfix “_choices.png” – will be produced, as well. The file with the postfix “_choices.png” will contain the image in which only the emblems and not the frames are presented.

**References**
Ooms, J. (2018). *magick:* *Advanced Graphics and Image-Processing in R.* R package version	1.9. https://CRAN.R-project.org/package=magick

Teetor, P. (2011). *R* *Cookbook*. Sebastopol, CA: O’Reilly Media, Inc.
